# Taxonomic and geographic bias in 50 years of research on the behaviour and ecology of galagids

**DOI:** 10.1371/journal.pone.0261379

**Published:** 2021-12-15

**Authors:** Grace Ellison, Martin Jones, Bradley Cain, Caroline M. Bettridge

**Affiliations:** Department of Natural Sciences, Manchester Metropolitan University, Manchester, United Kingdom; Instituto Federal de Educacao Ciencia e Tecnologia Goiano - Campus Urutai, BRAZIL

## Abstract

Identifying knowledge gaps and taxonomic and geographic bias in the literature is invaluable for guiding research towards a more representative understanding of animal groups. Galagids are nocturnal African primates and, for many species, detailed information on their behaviour and ecology is unavailable. To identify gaps and bias in the literature we reviewed published peer-reviewed research articles on galagid behaviour and ecology over a 50-year period from January 1971 to December 2020. Using the Web of Science and Google Scholar databases, we identified 758 articles, assessed 339 full texts for eligibility and included 211 in the review. Species of *Otolemur* have been extensively researched in comparison to other genera (78.2% of studies; *Euoticus*: 13.3% of studies; *Galago*: 66.4% of studies; *Galagoides*: 20.9% of studies; *Paragalago*: 22.3% of studies; *Sciurocheirus*: 15.2% of studies). The most common category of research was physiology (55.0% of studies), followed by behavioural ecology (47.4% of studies), and fewer studies were on genetics and taxonomy (16.1% of studies) and habitat and distribution (14.2% of studies). Text mining revealed that the word ‘behaviour’ was the most common word used in abstracts and keywords, and few words were related to ecology. Negative binomial regression revealed that mean body mass and geographic range size were significant positive predictors of the total number of scientific outputs on each species. Research on wild populations was carried out in only 24 (60%) of the 40 countries galagids are thought to inhabit. Studies were undertaken in locations with lower mean annual temperatures and higher human population densities over warmer and less populated areas. We encourage a more equal sampling effort both taxonomically and geographically that in particular addresses the paucity of research on smaller species and those with restricted ranges. Research on *in situ* populations, especially in warmer and remote areas, is urgently needed, particularly in West, Central and some Southern African countries.

## Introduction

We are losing species worldwide at such an alarming rate that we may be in the midst of the sixth mass extinction [1]. Species or populations lacking data on their behaviour and ecology are likely to be poorly understood and could be at greater risk of ‘silent’ extinction, where they may be overlooked by conservation management due to data deficiency and more likely to go extinct unnoticed [2, 3]. Despite this, research effort in animal behaviour and ecology is often dominated by a focus on certain taxonomic groups [4] and geographical study areas or biomes [5, 6]. Systematic reviews highlight the importance of evaluating the literature to quantify research outputs, reveal taxonomic or spatial biases, and identify areas in particular need of research [5, 7, 8].

Reviews have revealed taxonomic bias (when organisms from a particular taxonomic group are researched disproportionately to others) in studies across a wide range of biological disciplines [9, 10] as well as a lack of species-specific data in subjects such as animal behaviour [4] and conservation biology [7, 8, 11–14]. Within vertebrates, mammals and birds receive more research attention than any other group relative to their number of species [15, 16]. However, even within these well studied groups there can be imbalances, for example within European bird studies there is a strong focus on certain species [17], and the same has been found within studies on Australian terrestrial mammals [9] and Neotropical primates [18].

Research effort into larger species is generally far greater than that into smaller species (e.g. felids: [19, 20]; canids: [20]; carnivores: [21]; sharks: [22]; terrestrial mammals: [14]; Neotropical primates: [18]; Australian birds: [23]), and for species with larger geographic ranges than those with smaller ranges (Neotropical primates: [18]; carnivores: [21]; Australian birds: [23]; sharks: [22]). Population size [17] and habitat type [23] were important factors explaining research effort in ornithological studies, and in felids and canids the likelihood of being a keystone species was strongly positively correlated with research effort [20]. A global analysis of non-marine mammals showed that introduced species had a greater number of outputs than native species [14], and in some taxa aesthetics can also play a role; one study found that ‘ugly’ native eutherian species were studied less than native monotremes and marsupial species [9].

In addition to taxonomic bias, geographic bias also pervades the literature in animal behaviour and ecology [5, 6, 13, 24–26] and conservation biology [12, 27]. Sampling effort in ecological research is often biased towards areas that are easily accessible to humans, such as near rivers [5], cities, roads and other urban areas [5, 6, 23, 24]. For example, in Australia, records of koala presence closely mapped the road network [28]. Furthermore, researchers of tropical coral reefs favoured sites in wealthy nations near top-ranking research institutions over those with greater species richness [27]. Conservation priority areas, national parks and other protected areas are also used as study sites more than other areas [5, 6, 24, 29]. Alternatively, biologists may avoid using certain areas such as those affected by political instability and ongoing conflict [30, 31].

The majority of primate populations are threatened with extinction [32] and, like other animal groups, information on their behaviour and ecology is instrumental in the understanding of their conservation biology [33]. Although better-studied than most other mammalian groups [34], there are known biases in the research effort on primates. In recent years, primatologists showed a geographic bias for national parks and protected areas, using them as study sites in the vast majority of publications (73.3% [29]). Studies on parasites in wild primates are far more abundant on populations in East and South Africa than the rest of the world [30]. There is a taxonomic bias for specific groups, for example the great apes have been studied far more than other apes [35]. A recent review found that between 2011 and 2015, the three non-human great ape genera (*Pan*, *Pongo* and *Gorilla*) were in the top ten most studied genera of primates [29]. *Pan* and *Macaca* species have been studied at a far higher rate than all other primate genera, and no nocturnal primates featured at all [29]. Furthermore, a review of Neotropical primate diet studies found very few on nocturnal primates (night monkeys, *Aotus spp*: [18]), and compared to diurnal primates, nocturnal species are underrepresented in scientific documentaries and films [36].

Our review focuses on galagids, or ‘bushbabies’ (family: Galagidae); small, nocturnal, arboreal strepsirhine primates distributed throughout sub-Saharan Africa. Due to their cryptic morphology and nocturnal lifestyles, nocturnal strepsirhines were misclassified as just a few species for many years, but advances in their study revealed an incredibly diverse group of animals with varied social systems, locomotion and life histories [37]. Galagids are a useful model study group for understanding our earliest primate ancestors, which likely shared similar traits such as being small in size [38] and nocturnal [39]. Galagid behaviour and ecology varies greatly even within genera [40–42], and therefore extrapolating findings across even closely related species may be misleading and misinform conservation efforts.

Our aim was to systematically review and quantify the available literature on galagid behaviour and ecology from the last 50 years to identify the level of disparity in research effort among galagid species and choice of study locations. Specifically, our objectives were to investigate taxonomic bias in the total number of scientific outputs per species, and geographic bias in the study of free-ranging populations, as well as identify the types of samples used (wild, captive, museum-type, bioinformatic, or unknown) and topics of behaviour and ecology most researched.

We expected species with a greater body mass and larger geographic range to have more publications than smaller, range-restricted species, as seen in Neotropical primate studies [18], and expected areas with a greater human population density to be used as study sites more than less populated areas. We hypothesized that areas with lower mean annual temperatures would be preferred as study locations for logistical reasons such as increased accessibility and being less physiologically demanding for researchers. Similarly, we expected areas with lower mean annual rainfall to be popular as study sites, with the view that less dense vegetation would allow greater visibility and accessibility.

## Methods

### Note on taxonomy

We follow the taxonomy of Svensson *et al*. [41], who, in addition to the species recognised by Nekaris [43], used the genus name ‘*Paragalago*’ for the eastern clade of dwarf galagids [44], and included *Sciurocheirus makandensis* [45] and the recently described *Galagoides kumbirensis* [46]. Consistent with Svensson *et al*. [41], we hereafter use the abbreviation ‘*G*.’ for the genus *Galago* and ‘*Gd*.’ for *Galagoides* to avoid confusion. We therefore recognise the following 20 extant species of galagid: *Euoticus elegantulus*; *E*. *pallidus*; *Galago gallarum*; *G*. *matschiei*; *G*. *moholi*; *G*. *senegalensis*; *Galagoides demidovii*; *Gd*. *kumbirensis*; *Gd*. *thomasi*; *Otolemur crassicaudatus*; *O*. *garnettii*; *Paragalago cocos*; *P*. *granti*; *P*. *orinus*; *P*. *rondoensis*; *P*. *zanzibaricus*; *Sciurocheirus alleni*; *S*. *cameronensis*; *S*. *gabonensis*; and *S*. *makandensis*.

### Data compilation

We reviewed the available peer-reviewed research articles on the behaviour and ecology of galagids using a systematic approach. We largely adhered to the guidelines of Pullin and Stewart [47], ensuring that data were searched for, selected, extracted and evaluated systematically to allow for replication. However, to avoid pseudoreplication and ensure that our methods are replicable, we only included published peer-reviewed research papers [4, 10, 12, 20, 23, 27, 48] and excluded unpublished data (e.g. meeting abstracts, contacting experts in the field [49]). We completed the search in January 2021 on the Web of Science database, for publications from January 1971 to December 2020, using the following search terms: galag* AND: behav*; activity; social*; ecolog*; habitat; sleep*; feeding; distribution. The wildcard ‘galag*’ was also replaced by ‘bushbab*’ and ‘bush bab*’ for each of the 8 searches. Because galagids are African primates and the Web of Science does not support several African peer-reviewed scientific journals, we conducted a further search on the Google Scholar database using the ‘Advanced search’ tool, specifying that the word ‘African’ must be in the journal name. Google Scholar does not recognise wildcard searches (the use of asterisks to search for a word with the stated letters and any suffix) so we spelt all words out in full (all search terms are in S1 Table). We included articles published online or in print between, and including, 1^st^ January 1971 and 31^st^ December 2020.

### Data analysis

We initially screened the literature and included any records that had any of the following target words in the title, abstract or keywords: ‘bush baby’; ‘bush babies’; ‘bushbaby’; ‘bushbabies’; ‘galagid’; ‘galagids’; ‘Galagidae’; ‘galago’; ‘galagos’; and the previously used family name ‘Galagonidae’, and ‘Galagonid’ and ‘Galagonids’ (Jenkins, 1987, in Grubb *et al*. [50]). We removed duplicate papers that appeared in more than one search.

We read all articles and only included those with primary data on galagids to avoid pseudoreplication. We excluded papers that contained data on galagids, but did not contribute towards the understanding of their behaviour and ecology, often where researchers used them as models for other areas of research (e.g. functional neuroscience or gene function). For studies containing data on several species, we only recorded the information related to galagids.

We recorded the following information from each paper: date of publication; species; sample type (captive, wild, museum-type, bioinformatic [from bioinformatics databases, such as GenBank from the National Center for Biotechnology Information], or unknown); study location(s) with coordinates if available; the country of each authors’ affiliation(s) at the time of conducting the research; and categories of behaviour and ecology studied. Categories of behaviour and ecology were difficult to determine, as so many overlap [51], so we used four broad categories: behavioural ecology; habitat and distribution; physiology; and genetics and taxonomy. Some papers contributed to more than one area. For species that have had their taxonomy revised during the 50-year period, we used the location of the study site and geographic ranges to determine the current species name for studies on wild populations (e.g. between *G*. *senegalensis* and *G*. *moholi* [was *G*. *senegalensis moholi*]). Study locations were those where researchers studied free-ranging galagids; we did not record the origin of museum specimens or captive samples. For studies on other sample types, it was not possible to distinguish changes in taxonomy. We classed any studies on ‘*O*. *montieri*’ as *O*. *crassicaudatus* [52], and any on ‘*G*. *alleni*’ as *S*. *alleni* [53]. We classed all eastern *Gd*. spp. under their new recognised genus *Paragalago* [44]; this includes ‘*G*. *udzungwensis’* or ‘*Gd*. *udzungwensis*’ now being classed as *P*. *zanzibaricus* [54] and *Gd*. *nyasae* as *P*. *granti* [55].

#### Taxonomic bias

We investigated body mass, as a proxy for body size [14, 18–23], and geographic range size [18, 21–23] as potential drivers of taxonomic bias. To investigate what characteristics represented well-studied compared to poorly-studied species, we used negative binomial regression models to model the log of the expected total number of research outputs per species as a function of covariates: body mass and geographic range size. We used negative binomial regression rather than poisson regression for count data due to over-dispersion [56]. In addition to modelling the total number of research outputs per species, we used the same covariates to model the number of research outputs on wild populations only. We used separate models for total research and research only on wild populations instead of one model with random effects due to the small sample size (*N* = 17).

We used mean body mass data (g) from Butynski *et al*. [57] as a covariate. Mean body mass data were for males and females combined, and we could not find any available body mass data for *G*. *gallarum*, *Gd*. *kumbirensis* or *S*. *makandensis;* these three species were not included in the models.

We downloaded data on geographic range size for each species from the IUCN Red List (downloaded on 17^th^ August 2020 from https://www.iucnredlist.org) and combined any subspecies ranges into one for each species. We projected the ranges using the Africa Equal Area Conic projection to obtain one value for the area covered by each species in km^2^.

There was no substantial collinearity between body mass and geographic range size (*r*_*s*_ = 0.21, *P* = 0.417). We included years since scientifically recognised as an offset in the models because *P*. *rondoensis* was formally described in 1996 (Honess 1996, in Butynski *et al*. [57]) and the other 16 species were known to science for the whole sampling period. We report the McFadden’s pseudo *R*^*2*^ for our best model and for each covariate: the coefficient; standard error; *z*-statistic; *P*-value; 95% confidence intervals (95% CI); incident rate ratio; and 95% CI for the incident rate ratio.

#### Geographic bias

We created a map showing the number of studies published on galagid behaviour and ecology using wild samples from each country. We overlaid the locations of the populations studied and for papers where researchers used several study sites, we represented each study site separately on the map and in the models. If coordinates were not available in the paper, we entered the study site name into Google Earth (version 7.3.0.3830) and took the coordinates for the point chosen by the search (usually the mid-point) to include in the map. To investigate preference for study site locations we created an equal number of random points as there were study sites (*N* = 171) across the combined geographic range of all galagids and used logistic regression models with location (study site or random) as the dependent variable. We used data on local human population density as a measure of accessibility [5, 6, 23, 24]. We also investigated mean annual temperature, elevation and rainfall, as potential factors in choosing study locations. We projected all locations to the Africa Equal Area Conic projection, and created a 100 km buffer around the study sites and random locations to assess environmental variables in the areas surrounding study sites [18].

We downloaded human population density (humans/km^2^) raster data from near the mid-point of the survey period (2000) from the Socioeconomic Data and Applications Center (SEDAC; https://sedac.ciesin.columbia.edu/gpw; 1 x 1 km resolution; on 9^th^ September 2020). We also investigated mean annual temperature, elevation and rainfall, as potential factors in choosing study locations. We downloaded raster data for each covariate (mean annual temperature [°C]; annual precipitation [ml]; and digital elevation [m]) for the years 1970–2000 from WorldClim (https://www.worldclim.org; 1 x 1 km resolution; on 9^th^ September 2020). We projected all rasters to the Africa Equal Area Conic projection and calculated the mean value for each of the buffers to use in analysis. Mean annual temperature and mean elevation were highly negatively correlated (*R* = -0.815) so we only used mean annual temperature in the analysis. We used logistic regression models with location (study site or random) as the dependent variable and mean annual temperature, mean precipitation and mean human population density as covariates. We chose the model with the lowest AIC score as the best model and report the performance statistics (McFadden’s pseudo *R*^*2*^ and associated *P*-value) for the model and for each covariate: the coefficient; standard error; 95% CI; *z*-statistic; *P*-value; odds ratio; and 95% CI for the odds ratio.

#### Topics of behaviour and ecology studied

We used broad categories of behaviour and ecology (behavioural ecology; habitat and distribution; physiology; and genetics and taxonomy) to provide a general overview of the types of studies, but also identified specific topics researched within those categories. To highlight the most common topics studied we conducted text mining using the package ‘tm’ in R [58] to extract the 30 most common words used in abstracts and keywords, including any abbreviations stated below abstracts. We converted all text to lower case and removed all variants of ‘galagid’, ‘bushbaby’, species and genera names, and any other words not related to behaviour or ecology. We removed all punctuation, white space and numbers. We stemmed words to avoid repetition (e.g. ‘social’ combines ‘social’, ‘sociality’ and ‘socially’) and for each of the 30 most used stemmed words we include the un-stemmed words in S2 Table. If any of the top 30 stemmed words used groupings that were not semantically similar, we separated them. This was the case for two stemmed words; we split ‘activ’ into ‘active’ (active / activities / activity / activity-dependent) and ‘activate’ (activate / activated / activating / activation / activations), and ‘later’ into ‘later’ (later) and ‘lateral’ (lateral / lateralis / lateralised / laterality / lateralization / lateralized). We carried out all statistical tests in R version 4.0.3 [59]. Maps throughout this article were created using ArcGIS® software by Esri. ArcGIS® and ArcMap™ are the intellectual property of Esri and are used herein under license. Copyright © Esri. All rights reserved. For more information about Esri® software, please visit www.esri.com.

## Results

### Article inclusion

**Fig 1:**
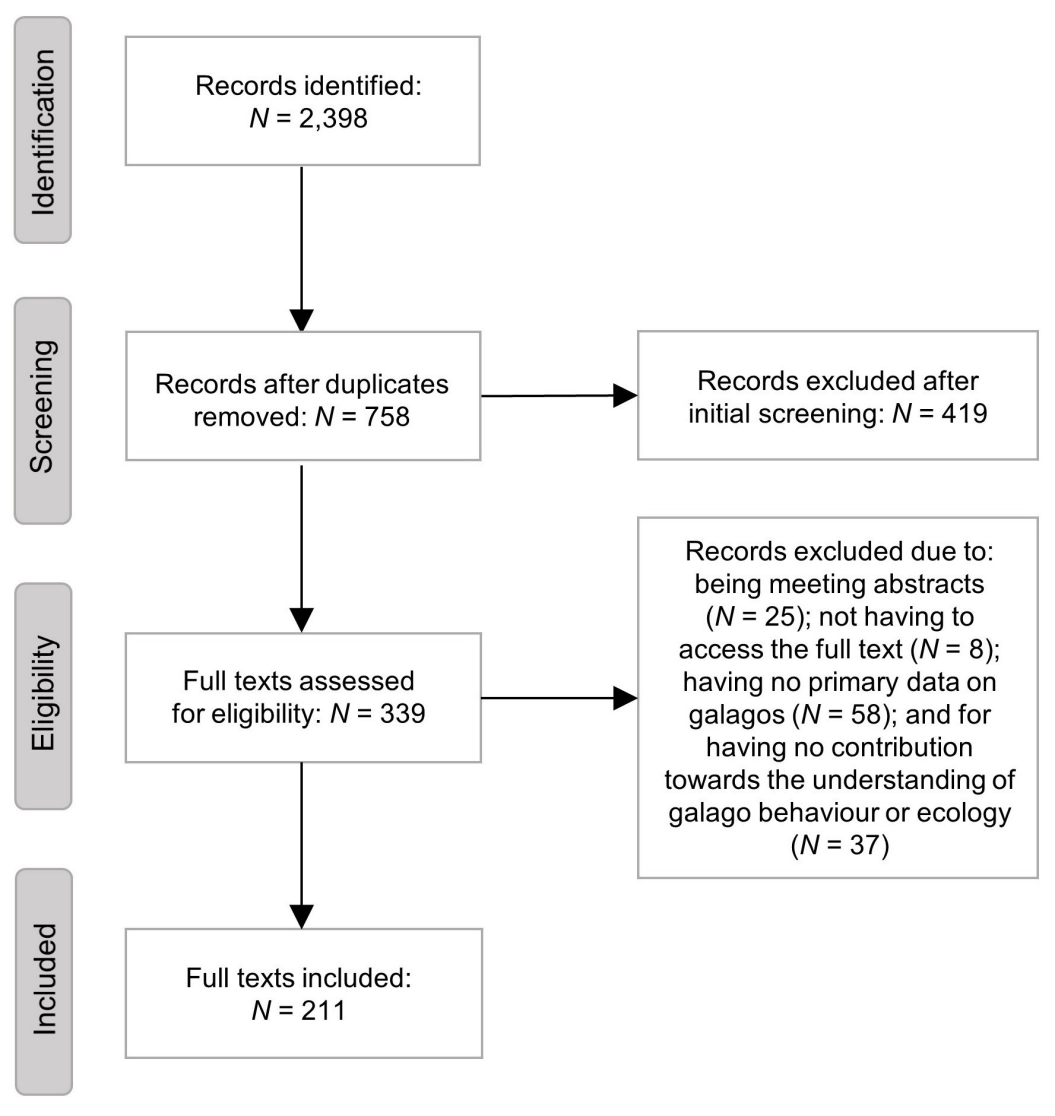
The screening process we used to review papers on galagid behaviour and ecology from January 1971 to December 2020.

**Fig 2:**
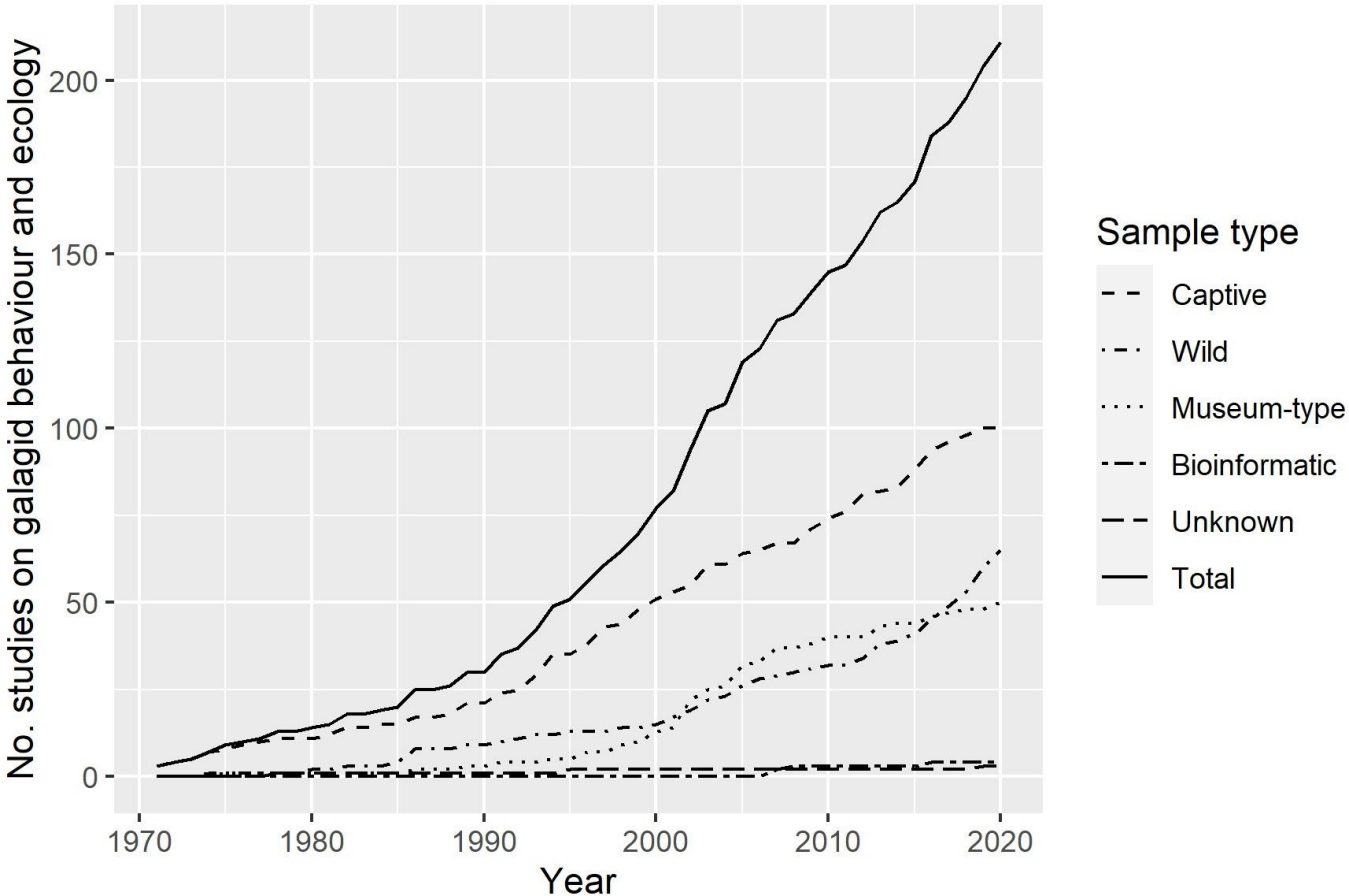
The number of research articles on galagid behaviour and ecology using captive, wild, museum-type, bioinformatic and unknown samples of galagids published between January 1971 and December 2020. The solid line denotes the cumulative number of studies.

The 96 search terms generated 2398 items in total (758 without duplicates; see S1 Table for breakdown of results for each search term). We included 339 based on one or more of our target words being present in the title (*N* = 179), abstract (*N* = 293) or the authors’ keywords (*N* = 107). Of the 339, we excluded 25 meeting abstracts, a further 8 because the full texts were not accessible, a further 58 articles for not including primary data on galagids and 37 more for having no contribution towards understanding the behaviour and ecology of galagids, leaving 211 for inclusion in the review (see Fig 1 for screening process, following Moher *et al*. [60]). There has been an increase in research into galagids from the late 1990s onwards (Fig 2) and the maximum number of studies published on galagid behaviour and ecology in any year (2016) was 13 (median = 4; IQR = 4.8).

### Author affiliations

Of the 211 studies, 63 (29.9%) were carried out by at least one author affiliated with an organisation based in a galagid range country. Of these 63, 36 involved international collaboration between researchers from galagid range countries and non-range countries. At least one author from a USA-based affiliation contributed to 132 studies (62.6%), at least one South African affiliation was connected to authors of 42 studies (19.9%) and at least one UK affiliation featured in 32 studies (15.2%). At least one author from an institution based in Germany contributed to 20 studies (9.5%) and affiliations from all other countries were connected to fewer than 10 studies.

### Sample type

There were far more studies on captive galagids than any other sample type (47.4%; *N* = 100). Data on wild individuals were included in 30.8% of studies (*N* = 65), 23.7% (*N* = 50) included data from museum-type specimens and very few used bioinformatic data (1.9%; *N* = 4). For 1.4% of studies (*N* = 3) it was unclear which type of sample was used. The number of research outputs on wild populations increased at a faster rate in the last 5 years than the years before (see Table 1 and Fig 2).

**Table 1:** The total number of research articles on galagid behaviour and ecology published between January 1971 and December 2020, the conservation status and reference to IUCN Red List web pages for each species.

Species	No. research outputs	IUCN status	Reference
*Euoticus elegantulus*	21	LC	[61]
*Euoticus pallidus*	6	NT	[62]
*Galago gallarum*	11	LC	[63]
*Galago matschiei*	11	LC	[64]
*Galago moholi*	61	LC	[65]
*Galago senegalensis*	55	LC	[66]
*Galagoides demidovii*	27	LC	[67]
*Galagoides kumbirensis*	3	NT	[68]
*Galagoides thomasi*	12	LC	[69]
*Otolemur garnettii*	85	LC	[70]
*Otolemur crassicaudatus*	78	LC	[52]
*Paragalago cocos*	5	LC	[71]
*Paragalago granti*	10	LC	[55]
*Paragalago orinus*	7	VU	[72]
*Paragalago rondoensis*	5	EN	[73]
*Paragalago zanzibaricus*	19	NT	[54]
*Sciurocheirus alleni*	26	NT	[53]
*Sciurocheirus cameronensis*	1	NE	-
*Sciurocheirus gabonensis*	3	LC	[74]
*Sciurocheirus makandensis*	2	DD	[45]

IUCN Red List abbreviations used above: ‘LC’ = least concern, ‘NT’ = near threatened; ‘VU’ = vulnerable; ‘EN’ = endangered; ‘DD’ = data deficient; ‘NE’ = not evaluated.

There is currently no IUCN Red List status for *Sciurocheirus cameronensis*.

### Taxonomic bias

**Fig 3:**
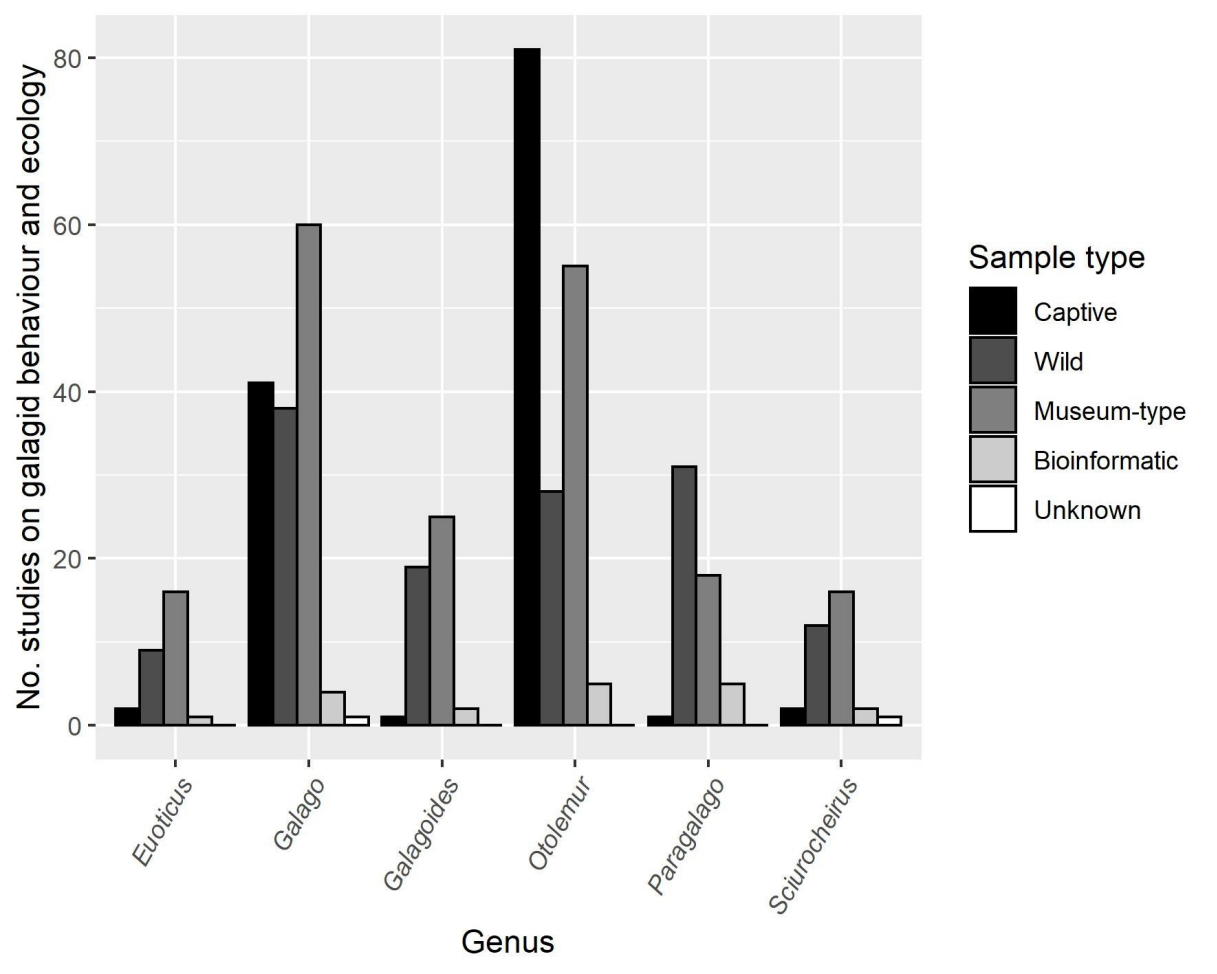
The number of research articles on captive, wild, museum-type, bioinformatic and unknown samples of each galagid genus published between January 1971 and December 2020.

Species within the genus *Otolemur* featured in the majority of research outputs, and far more than other species (78.2%, *N* = 165; *Euoticus*: 13.3%, *N* = 28; *Galago*: 66.4%, *N* = 140; *Galagoides*: 20.9%, *N* = 44; *Paragalago*: 22.3%, *N* = 47; *Sciurocheirus*: 15.2%, *N* = 32; these values include outputs on unknown species within genera—see Table 1 for total outputs per individual species). Thirteen species featured in fewer than 20 publications and eight species were studied fewer than ten times. The number of studies on each genus using different sample types is in Fig 3. Three species of *Sciurocheirus* (*S*. *cameronensis*, *S*. *gabonensis* and *S*. *makandensis*) were vastly underrepresented, with *S*. *cameronensis* and *S*. *makandensis* featuring in fewer articles than the recently described *Gd*. *kumbirensis* (described in 2017 by Svensson *et al*. [46]).

Our best negative binomial model to predict the total number of research outputs per species included both body mass (g) and geographic range size (1000 km^2^) (AIC = 137.17; McFadden’s pseudo *R*^*2*^ = 0.880; see Table 2) rather than body mass only (AIC = 143.73) or geographic range size only (AIC = 162.50). Incident rate ratios revealed that, holding other variables in the model constant, for every one unit increase in body mass (1 g) the total research output is expected to increase by a factor of 1.002 (95% CI = 1.001–1.003). Holding other variables in the model constant, for every one unit increase in geographic range size (1000 km^2^) the total research output is expected to increase by a factor of 1.0002 (95% CI = 1.0001–1.0004). Because of the high number of captive studies on *Otolemur* spp., we also ran the models with captive studies removed and both predictors were still significant in the best model (body mass: *P* = 0.027; geographic range size: *P* = 0.010). However, when only studies on wild populations were considered, neither body mass or geographic range size were significant predictors of the number of research outputs per species (see Table 2).

**Table 2:** Results from the negative binomial regression models used to investigate taxonomic bias in 50 years of research articles on galagid behaviour and ecology.

Dependent variable	Covariate	Coefficient	SE	95% CI	*z*	*P*-value
Total studies per species	Body mass^1^	0.002	6.04E-04	7.27E-04–0.003	3.137	0.002**
	Range size^2^	2.20E-04	6.97E-05	8.56E-05–3.70E-04	3.151	0.002**
Wild studies per species	Body mass^1^	5.84E-04	4.65E-04	-3.15E-04–0.002	1.256	0.209
	Range size^2^	9.60E-05	5.49E-05	-1.33E-05–2.09E-04	1.749	0.080

*Data sources*: *1*. [57]*; 2*. www.iucnredlist.org/

For each covariate we report the coefficient, standard error (SE), 95% confidence intervals (95% CI), z-statistic and approximate P-value.

### Geographic bias

**Fig 4:**
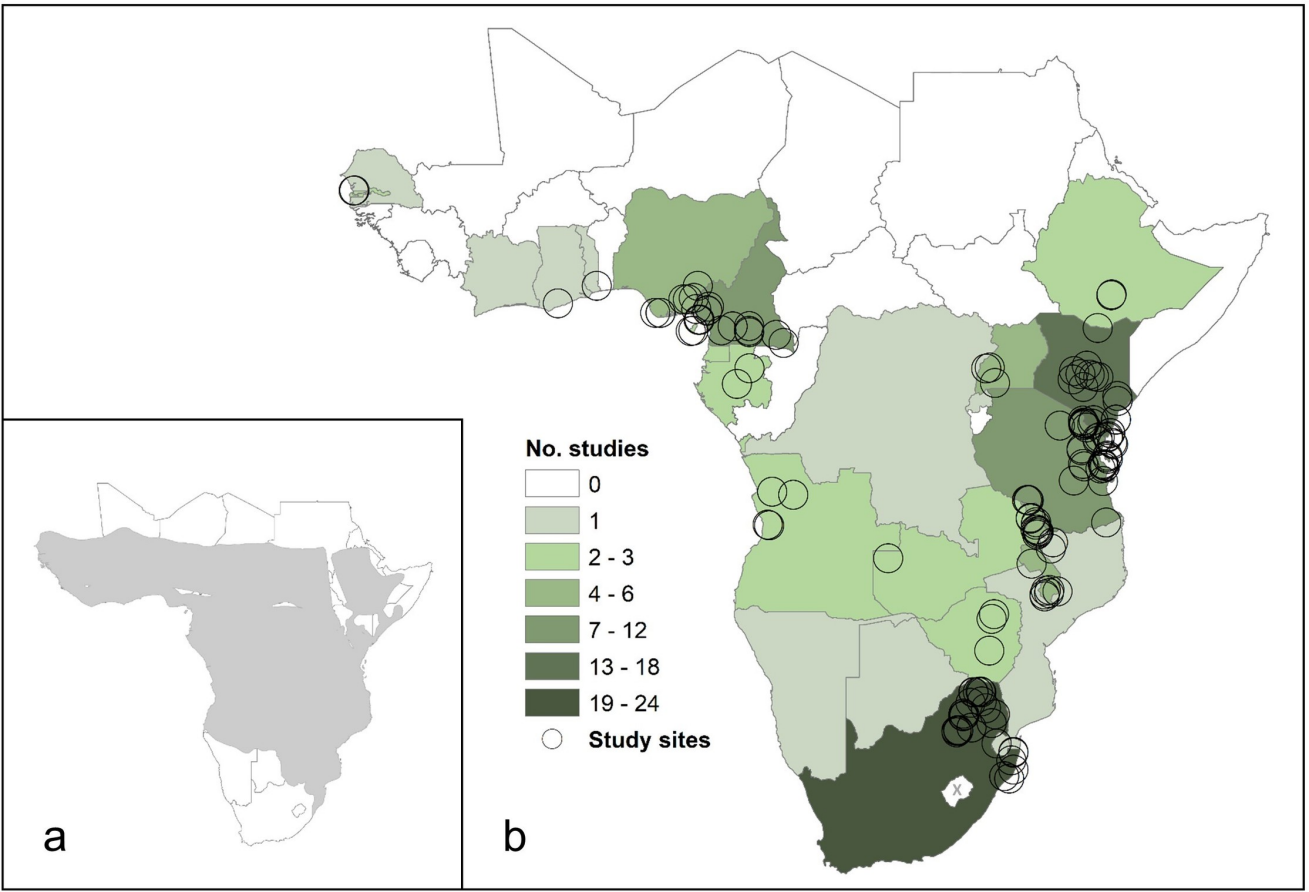
**a)** The combined geographic range of all galagids according to IUCN data. **b)** The number of research articles on galagid behaviour and ecology using wild samples from each country published between January 1971 and December 2020. White countries are those inhabited by at least one species of galagid according to IUCN geographic range data, but where no studies have yet been conducted. Lesotho is landlocked and therefore seen in the map above, marked with a grey cross because no free-ranging galagids are confirmed to be there based on IUCN data. South Sudan is also landlocked but the presence of one species, Gd. thomasi, is uncertain there [69] so we include it as a country galagids are thought to inhabit. The black circles mark the locations of study sites used to research galagids; for some studies there were several sites.

**Fig 5:**
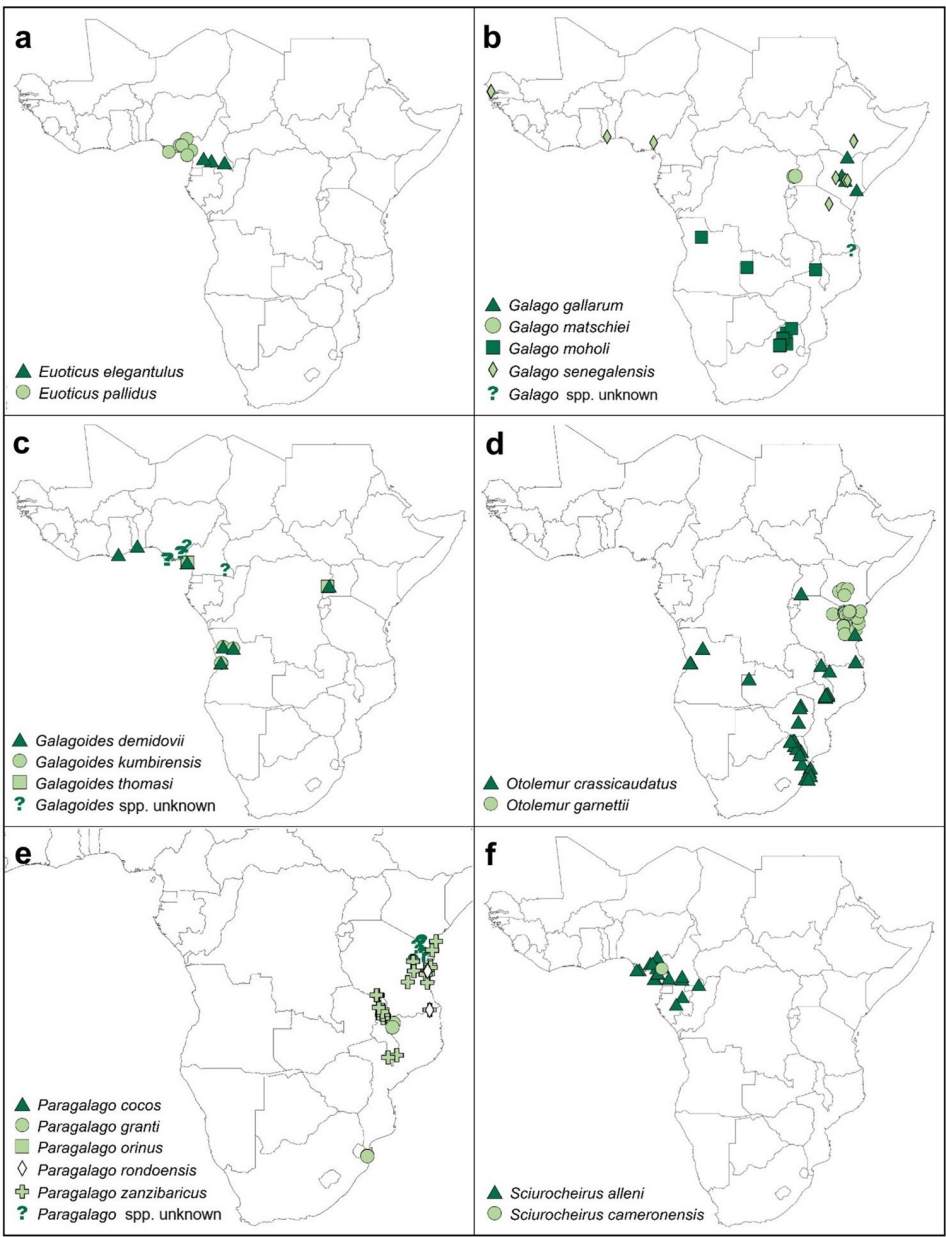
The location of study sites for each galagid species in research articles published between January 1971 and December 2020. We split any study sites for unknown dwarf galagid species into ‘Galagoides spp. unknown’ or ‘Paragalago spp. unknown’ based on the location and IUCN geographic range data.

The included studies spanned 24 countries in Africa (60% of countries galagids are thought to inhabit). Twenty-four of the 65 studies on free-ranging populations included research on galagids resident to South Africa; 15 in Kenya; 12 in Tanzania; nine in Cameroon; six in Uganda and Malawi; four in Nigeria; three in Angola and Equatorial Guinea; two in Ethiopia, Gabon, The Gambia, Zambia and Zimbabwe; and one in each of: Botswana, Cote d’Ivoire, the Democratic Republic of Congo, Eswatini, Ghana, Mozambique, Namibia, Rwanda, Senegal and Togo (see Fig 4 for the number of studies per country and the distribution of all study sites). No studies on wild galagids were conducted in 16 countries they are thought to inhabit according to IUCN geographic range data. These countries are: Benin; Burkina Faso; Burundi; Central African Republic; Chad; Congo; Eritrea; Guinea; Guinea-Bissau; Liberia; Mali; Niger; Sierra Leone; Somalia; South Sudan; Sudan. The distribution of study sites for each species are in Fig 5.

Our best logistic regression model to predict study locations included mean annual temperature and mean human population density as covariates (AIC = 426.50; McFadden’s pseudo *R*^*2*^ = 0.113; *P* <0.001; see S3 Table for comparison of model performance). Mean human population density (humans/km^2^; coefficient = 0.007; SE = 0.002; 95% CI = 0.004–0.011; *z* = 4.010, *P* <0.001) and mean annual temperature (°*C*; coefficient = -0.233; SE = 0.044; 95% CI = -0.322 –-0.148; *z* = -5.262; *P* <0.001) were significant predictors of galagid study locations compared to random locations. The odds ratio for mean annual temperature suggests that, holding other variables at a fixed value, for a one unit increase in temperature (1°*C*) the odds of that location being chosen as a study site decreases by 20.8% (odds ratio = 0.792; 95% CI = 0.724–0.862). The odds ratio for mean human population density suggests that, holding other variables at a fixed value, for a one unit increase in human population density (humans/km^2^) the odds of that location being chosen as a study site increases by 0.7% (odds ratio = 1.007; 95% CI = 1.004–1.011).

### Topics of behaviour and ecology

**Fig 6:**
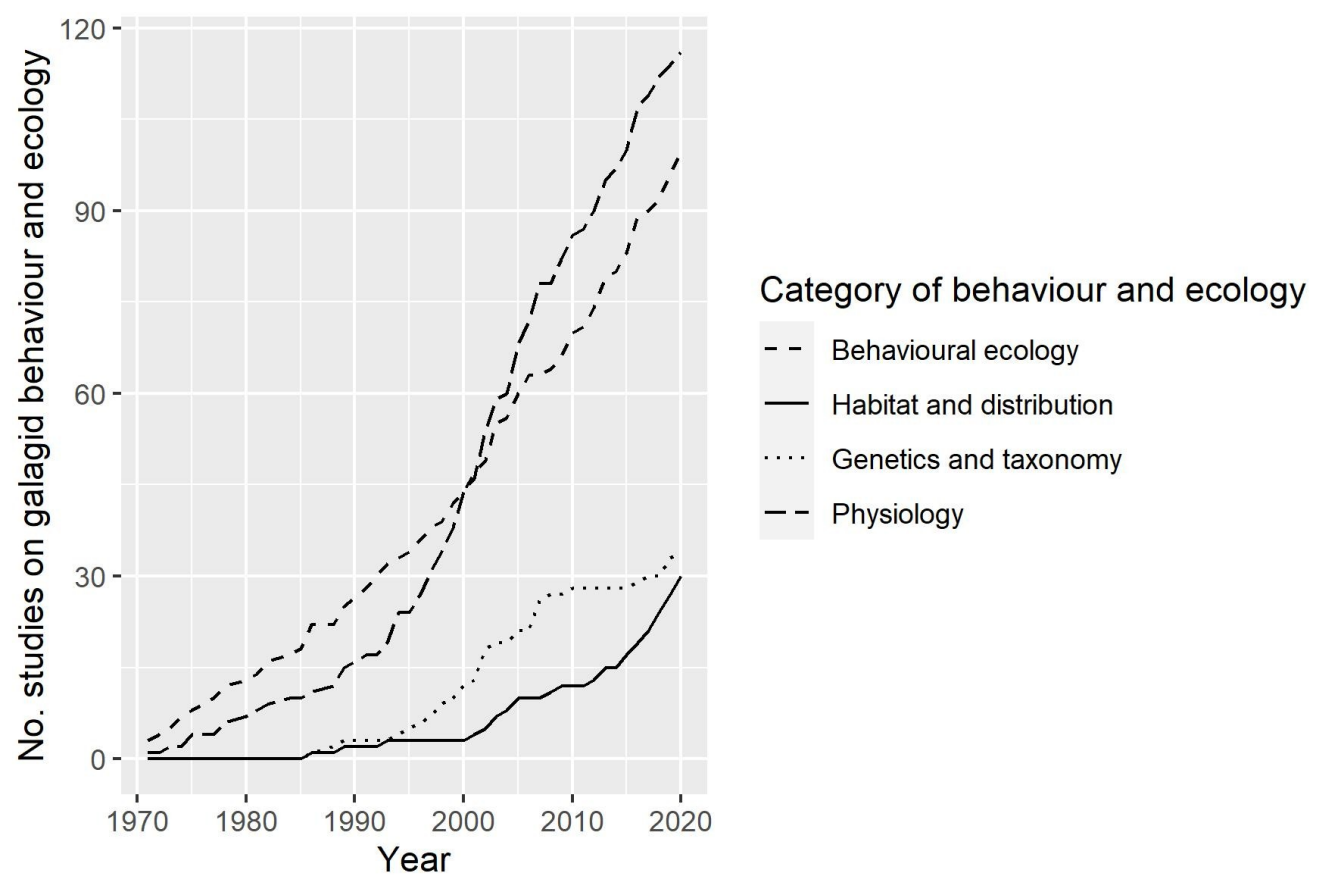
The number of research articles on each category of galagid behaviour and ecology published between January 1971 and December 2020.

Of the 211 publications, we classified 116 studies as contributing to the knowledge of galago physiology (55.0%), 100 to behavioural ecology (47.4%), 34 to genetics and taxonomy (16.1%) and 30 to habitat and distribution (14.2%; see Fig 6).

**Fig 7:**
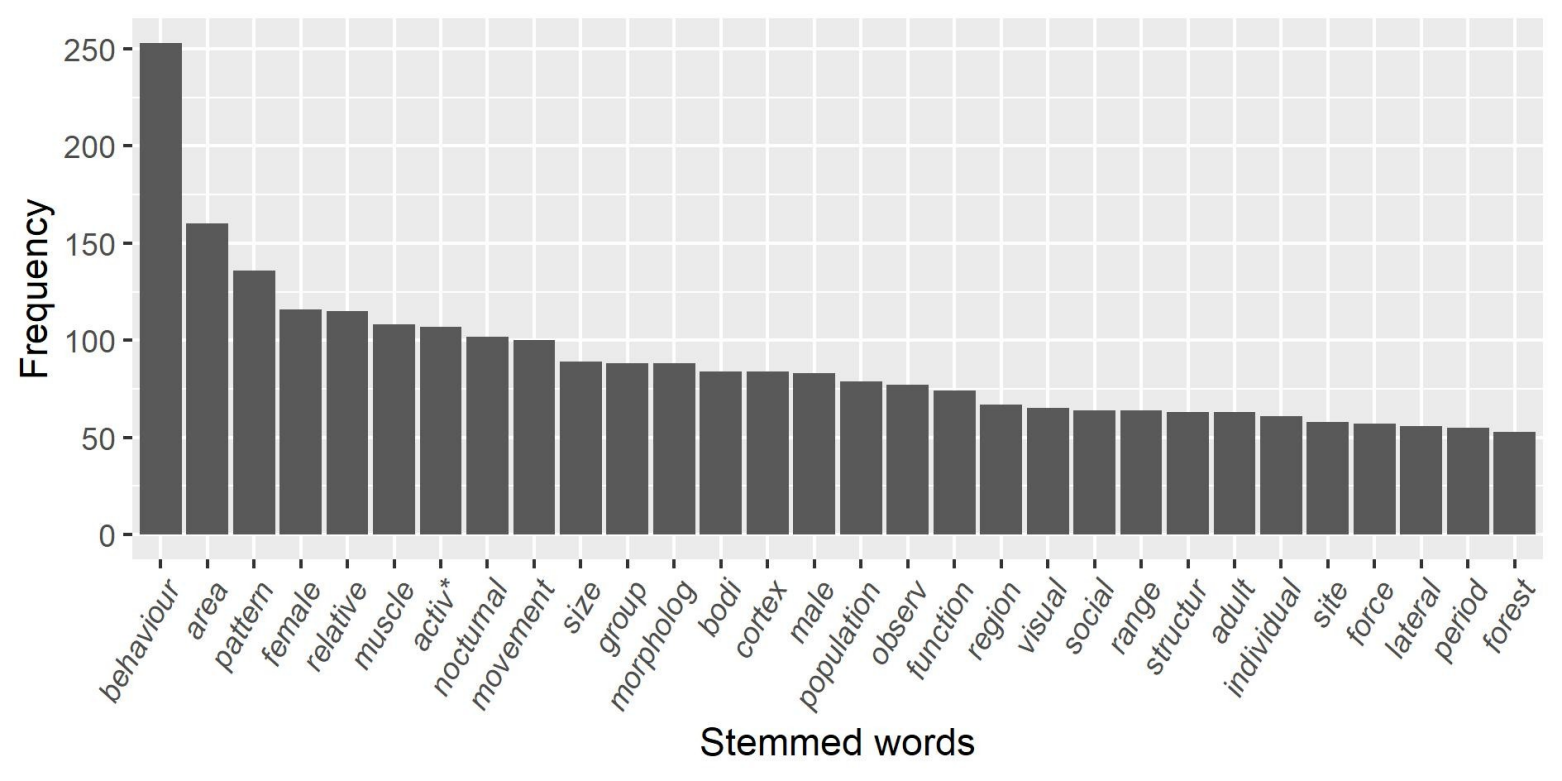
The 30 most used (stemmed) words in abstracts and keywords of research articles on galagid behaviour and ecology from January 1971 to December 2020. We excluded words not related to behaviour and ecology. *‘activ’ refers to the following words: ‘active’, ‘activities’, ‘activity’, ‘activity-dependent’; not: ‘activate’, ‘activated’, ‘activating’, ‘activation’, or ‘activations’.

Text mining revealed that the most common word used by researchers studying galagid behaviour and ecology was ‘behaviour’ (see Fig 7). Many words can refer to general areas of behaviour (e.g. ‘behaviour’, ‘activ*’, ‘social’), with some more focussed on particular topics such as locomotion (e.g. ‘movement’, ‘muscle’, ‘force’). Galagid appearance and physiology were commonly referred to (e.g. ‘morpholog’, ‘bodi’, ‘function’) with brain research being a popular topic of study (e.g. ‘cortex’, ‘region’, ‘lateral’).

## Discussion

In 50 years of research, 211 articles contributed to the understanding of galagid behaviour and ecology. The total research effort on galagids was not equally distributed among the species, and was generally higher for those with a greater body mass and larger geographic range; we know far less about smaller and range-restricted galagids. However, neither variable was a significant predictor when only the number of wild studies for each species were considered. Studies on galagids were more abundant in areas with a greater human population density and lower temperatures, suggesting that the behaviour and ecology of galagids in rural areas and those with higher temperatures may be poorly understood. Captive galagids were researched far more than wild populations or museum-type specimens, therefore many of the findings we have on galagids may not apply to wild populations.

### Taxonomic bias

Consistent with the literature on other animal groups [9, 15–18, 75], we found a taxonomic bias in the literature on galagid behaviour and ecology. The total research effort was generally greater for species with a larger body mass [14, 18–23] and larger geographic range [18, 21, 23]. Species of *Otolemur* were studied far more than any other species, particularly captive populations, but both body mass and geographic range size were still significant predictors of research output when captive studies were removed. However, this relationship did not persist when only studies on wild populations were considered. There are a number of possible explanations for this. We have not accounted for the number of each species that exist in captive facilities or museums, which could have influenced the results. If species with larger body masses are more abundant in captivity and museums, it may be because they have been spotted with greater ease in the wild, and consequently captured more for zoos and museums. *Otolemur garnettii* are known for their loud vocalisations [76], making them more conspicuous than other species, which could have resulted in the abundance of *Otolemur* in captivity. Larger species such as *O*. *garnettii* may be easier to keep in captivity owing to their varied diet [37] whereas specialists such as *E*. *elegantulus* [37], which rely more on tree gum, may be more difficult to provide for in captivity. A further possible explanation is that larger species are intrinsically more popular and charismatic to researchers. Larger bodied animals are favoured by visitors to zoos over smaller animals [77, 78], so it is possible that they were preferred model species by researchers, or that this knowledge encouraged captive facilities and museums to keep larger species that were therefore available for research.

For many animal species, geographic distribution and abundance are positively associated [79, 80], so species with larger geographic ranges may be more likely to be encountered. Our results suggest that species with larger geographic ranges have not necessarily had more studies on wild populations, but their larger spatial distributions have likely allowed a greater presence in captive facilities and museums than those with smaller ranges. It is important to note that the sample size (*N* = 17 species) was fairly small and a larger sample size, if available, would have increased the power of the models.

Understanding the behaviour and ecology of a species is essential for effective conservation management [81] and we cannot ignore the conservation implications that arise from taxonomic bias. Of the six species classified as NT, VU, and EN, four (*P*. *rondoensis*, *P*. *orinus*, *Gd*. *kumbirensis and E*. *pallidus)* featured in fewer than 10 articles. Owing to their extinction risks, we should prioritise research on these species for their conservation. *Sciurocheirus cameronensis* has not been assessed by the IUCN Red List and must be done so in the near future. Knowing so little about some galagid species has potentially devastating consequences for their conservation; understudied species may be at greater risk of extinction than those well studied, and could be more susceptible to ‘silent’ extinction [2, 3].

### Geographic bias

Geographic bias is present in the literature on galagid behaviour and ecology, as seen in other animal groups [5, 6, 13, 24–26, 29]. In 50 years of research, just under half of the countries galagids are thought to inhabit were not represented. The most common country visited to research them was South Africa, followed by Kenya, Tanzania and Cameroon. West Africa, Central Africa and some areas of Southern Africa (Namibia, Botswana and Mozambique), are in urgent need of research on galagids for us to understand the requirements of populations and obtain sufficient knowledge to conserve them.

Studies on wild galagids were generally carried out in areas with a greater human population density, suggesting that accessibility is an important factor in choosing a study site, likely due to the abundance of roads, cities and other urban areas [5, 6, 18, 23, 24]. Some of the countries with few or no published studies may be considered as difficult to travel to for foreign researchers or unsafe due to ongoing conflict [82, 83]. In Angola for example, research on galagids has only been possible in recent years following the end of the war [84]. It is also possible that researchers avoided areas for political reasons [30, 31]. Study site locations were in areas with cooler mean annual temperatures than random locations across the combined geographic range of all galagids. The combined range crosses the equator and stretches up to the southern end of the Sahara desert, supporting some of the highest temperatures in the world. This could explain why researchers of galagids preferred cooler sites that may not have posed as much physiological stress on the body as those in extremely hot areas. It would also be beneficial to confirm that galagids are present in the warmer and more remote areas of their proposed ranges, and to investigate the coping strategies they adopt to survive under warmer conditions.

Collaboration with local researchers could help expand research to the less-studied areas and species [85]. Less than one third (29.4%) of the studies on galagid behaviour and ecology were carried out by at least one author with an affiliation in a galagid range country. Just over half of those studies involved collaboration between researchers living in galagid range countries and those living in other parts of the world. Collaboration with local researchers can increase knowledge transfer and help to implement conservation policies [86]. We note that by using the countries where the institutions are based in this review, we did not account for the possibility that researchers from galagid range countries may be affiliated with organisations in non-range countries.

For a better understanding of galagid behaviour and ecology, we must ensure that the study locations are representative of their whole geographic range, where feasible and safe to do so. Geographic sampling bias could skew the biological knowledge necessary for conservation management [6, 26] and sampling previously unrepresented populations may lead to new discoveries about the behaviour of a species [5]. We hope that our results, particularly Figs 4 and 5, can guide future research towards less-visited study areas if a target species is in mind.

### Sample type

Although the greater galagos *O*. *crassicaudatus* and *O*. *garnettii* featured in the largest number of studies, most of those studies focussed on captive or museum-type samples rather than wild populations. We found a similar pattern with all other genera except for *Paragalago*, in which the majority of the studies were on wild populations. With primate populations threatened with extinction on a global scale [32] it is imperative that further research into the behaviour and ecology of *in situ* populations is carried out and that populations are monitored over the long term. We anticipate that bioinformatic data will be more popular to use with advances in genetics and technology. We note that, due to the many taxonomic revisions within the galagids, the number of studies on each species may not be entirely accurate, particularly for those using captive or museum-type samples.

### Categories and topics of behaviour and ecology studied

Physiology was the most common category of behaviour and ecology studied, followed by behavioural ecology. Galagid locomotion is of great interest [87–89], particularly the saltatory locomotion of the leapers [89–94], which may have increased the number of studies on their physiology substantially. Correspondingly, three of the most common words used by researchers in abstracts and keywords were ‘movement’, ‘muscle’ and ‘force’, highlighting the abundance of studies on galagid locomotion.

‘Behaviour’ was the most commonly used stemmed word and researchers used ‘activ’ and ‘social’ more than most other words. Galagids are generally thought to be solitary [95] but for decades researchers have highlighted elements of their social behaviour [37, 96–106], and the prominent use of the word ‘social’ is in accordance with this.

From the articles reviewed here, research on galagid habitat and distribution, and genetics and taxonomy, is much less frequent. Fewer studies contributed to the habitat and distribution category than any other category. Moreover, ‘ecology’, or a stemmed version of the word, was not one of the 30 most used words in abstracts and keywords. However, there has been a steady increase in studies on habitat and distribution in the last 20 years, following a similar pattern to the increase in the number of studies on wild populations, and some of the most commonly used words may indicate the study of wild populations (e.g. ‘population’, ‘range’, ‘area’, ‘region’). It is no surprise that taxonomic revisions within the Galagidae family have occurred in recent years and that further revisions are expected [42, 44, 46, 50] when so few studies have investigated their genetics and taxonomy. It is possible that our choice of search terms influenced the number of papers on genetics and taxonomy. We did not include the words ‘genetics’, ‘taxonomy’ and ‘physiology’ as search terms because these were not the focus of our review, but this is unlikely to have biased the results because ‘physiology’ was still the most common category of behaviour and ecology studied.

A possible limitation with this review is that it does not include books and book chapters, without which we would know far less about this diverse group of animals. These types of sources often include overviews, summaries, and collated knowledge from researchers on the taxonomy, physiology, behaviour and conservation of each species [37, 43, 57, 107, 108]. Others investigated the behaviour and ecology of particular species of galagid in greater depth. For example, Charles-Dominique [109] described the ecology and behaviour of galagids (*E*. *elegantulus*, *S*. *alleni* and *G*. *demidovii*) in Gabon in detail, including how these species live sympatrically along with two other nocturnal primates (pottos: *Perodicticus potto*; and Calabar angwantibos: *Arctocebus calabarensis*) by occupying separate ecological niches within the forest. The influence of predators on foraging in *G*. *moholi* is compared to that of the grey slender loris (*Loris lydekkerianus*) in Bearder *et al*. [110].

There may also be some studies we did not find due to our chosen search criteria. Some may not be published due to issues such as small sample size, non-significant results, or other methodological handicaps [10], but could have contributed to our knowledge on galagids. Due to known taxonomic bias within the Primate family [29] it is possible that there is a publication bias, with studies on certain groups favoured over galagids. Galagid research may also be reported in the grey literature, on websites, in languages other than English and in technical reports. However, we did not find any evidence of technical reports or studies in non-English languages during our literature search. For future research, it would be interesting to investigate whether the books and any other sources on galagid behaviour and ecology support or contradict the findings in this review.

## Conclusion

Taxonomic and geographic bias in research effort skews our knowledge of animal behaviour and ecology, presenting challenges for conservation. In the case of the galagids, research is urgently needed on the smaller and range-restricted species. Further research on galagid behaviour and ecology is needed across most of sub-Saharan Africa, and urgently in West, Central and some Southern African countries. Logistical and financial constraints can understandably compromise the questions researchers are able to answer and the animals they can study to answer those questions. Unfortunately, as a result, our understanding of animal behaviour and ecology is skewed and limited, but there is potential for a wealth of new discoveries. Researchers may inevitably continue to show bias for particular species or study sites, but we hope that our review can act as a guide to direct future research on galagids and alleviate some of the biases found here.

## Supporting information

S1 Table Search terms used in Web of Science and Google Scholar to find articles on galagid behaviour and ecology published between January 1971 and December 2020. For the Google Scholar search, we specified to search only journals with the word ‘African’ in the journal name.
(DOCX)

S2 Table The 30 most common stemmed words and corresponding un-stemmed words relating to galagid behaviour and ecology, used in scientific papers from January 1971 and December 2020. (DOCX)

S3 Table Comparison of model performance (AIC) of logistic regression models used to investigate geographic bias in the locations of study sites used to research galagids between January 1971 and December 2020. Covariates are: mean annual temperature (‘temperature’;°C); mean human population density from the year 2000 (‘human population density’; humans/km^2^); and mean annual precipitation (‘precipitation’; ml). Our best model is in bold.
(DOCX)
