# Supplementary material for: Taxonomic and geographic bias in 50 years of research on the behaviour and ecology of galagids
Source: PLoS One. 2021 Dec 15;16(12):e0261379. doi: 10.1371/journal.pone.0261379 (PMC8673608; doi:10.1371/journal.pone.0261379)
Supplement: S1 Table — For the Google Scholar search, we specified to search only journals with the word ‘African’ in the journal name. (DOCX) [file pone.0261379.s001.docx]

***Supplementary Table 1.*** *Search terms used in Web of Science and Google Scholar to find articles on galagid behaviour and ecology published between January 1971 and December 2020. For the Google Scholar search, we specified to search only journals with the word ‘African’ in the journal name.*

| **Search engine** | **Search term** | **No. results** |
| --- | --- | --- |
|  |  |  |
| Web of Science | galag* AND behav* | 182 |
| Web of Science | bushbab* AND behav* | 54 |
| Web of Science | bush bab* AND behav* | 38 |
| Web of Science | galag* AND activity | 104 |
| Web of Science | bushbab* AND activity | 24 |
| Web of Science | bush bab* AND activity | 10 |
| Web of Science | galag* and social* | 59 |
| Web of Science | bushbab* and social* | 16 |
| Web of Science | bush bab* and social* | 15 |
| Web of Science | galag* AND ecolog* | 48 |
| Web of Science | bushbab* AND ecolog* | 10 |
| Web of Science | bush bab* AND ecolog* | 11 |
| Web of Science | galag* AND habitat | 46 |
| Web of Science | bushbab* AND habitat | 15 |
| Web of Science | bush bab* AND habitat | 9 |
| Web of Science | galag* AND sleep* | 13 |
| Web of Science | bushbab* AND sleep* | 3 |
| Web of Science | bush bab* AND sleep* | 1 |
| Web of Science | galag* AND feeding | 37 |
| Web of Science | bushbab* AND feeding | 11 |
| Web of Science | bush bab* AND feeding | 7 |
| Web of Science | galag* AND distribution | 101 |
| Web of Science | bushbab* AND distribution | 14 |
| Web of Science | bush bab* AND distribution | 12 |
|  |  |  |
| Google Scholar | galago and behaviour | 98 |
| Google Scholar | galago AND behavior | 50 |
| Google Scholar | galagos AND behaviour | 20 |
| Google Scholar | galagos AND behavior | 18 |
| Google Scholar | bushbaby AND behaviour | 21 |
| Google Scholar | bushbaby AND behavior | 12 |
| Google Scholar | bushbabies AND behaviour | 21 |
| Google Scholar | bushbabies AND behavior | 14 |
| Google Scholar | bush baby AND behaviour | 11 |
| Google Scholar | bush baby AND behavior | 7 |
| Google Scholar | bush babies AND behaviour | 13 |
| Google Scholar | bush babies AND behavior | 5 |
| Google Scholar | galago AND activity | 121 |
| Google Scholar | galagos AND activity | 18 |
| Google Scholar | bushbaby AND activity | 15 |
| Google Scholar | bushbabies AND activity | 16 |
| Google Scholar | bush baby AND activity | 10 |
| Google Scholar | bush babies AND activity | 11 |
| Google Scholar | galago AND social behaviour | 11 |
| Google Scholar | galago AND social behavior | 4 |
| Google Scholar | galagos AND social behaviour | 1 |
| Google Scholar | galagos AND social behavior | 3 |
| Google Scholar | bushbaby AND social behaviour | 3 |
| Google Scholar | bushbaby AND social behavior | 1 |
| Google Scholar | bushbabies AND social behaviour | 3 |
| Google Scholar | bushbabies AND social behavior | 1 |
| Google Scholar | bush baby AND social behaviour | 1 |
| Google Scholar | bush baby AND social behavior | 1 |
| Google Scholar | bush babies AND social behaviour | 1 |
| Google Scholar | bush babies AND social behavior | 0 |
| Google Scholar | galago AND sociality | 4 |
| Google Scholar | galagos AND sociality | 4 |
| Google Scholar | bushbaby AND sociality | 2 |
| Google Scholar | bushbabies AND sociality | 2 |
| Google Scholar | bush baby AND sociality | 1 |
| Google Scholar | bush babies AND sociality | 1 |
| Google Scholar | galago AND ecology | 121 |
| Google Scholar | galagos AND ecology | 37 |
| Google Scholar | bushbaby AND ecology | 29 |
| Google Scholar | bushbabies AND ecology | 29 |
| Google Scholar | bush baby AND ecology | 10 |
| Google Scholar | bush babies AND ecology | 11 |
| Google Scholar | galago AND ecological | 93 |
| Google Scholar | galagos AND ecological | 32 |
| Google Scholar | bushbaby AND ecological | 20 |
| Google Scholar | bushbabies AND ecological | 22 |
| Google Scholar | bush baby AND ecological | 11 |
| Google Scholar | bush babies AND ecological | 10 |
| Google Scholar | galago AND habitat | 101 |
| Google Scholar | galagos AND habitat | 35 |
| Google Scholar | bushbaby AND habitat | 27 |
| Google Scholar | bushbabies AND habitat | 24 |
| Google Scholar | bush baby AND habitat | 13 |
| Google Scholar | bush babies AND habitat | 10 |
| Google Scholar | galago AND sleeping site | 9 |
| Google Scholar | galagos AND sleeping site | 7 |
| Google Scholar | bushbaby AND sleeping site | 3 |
| Google Scholar | bushbabies AND sleeping site | 6 |
| Google Scholar | bush baby AND sleeping site | 1 |
| Google Scholar | bush babies AND sleeping site | 4 |
| Google Scholar | galago AND feeding | 59 |
| Google Scholar | galagos AND feeding | 18 |
| Google Scholar | bushbaby AND feeding | 11 |
| Google Scholar | bushbabies AND feeding | 15 |
| Google Scholar | bush baby AND feeding | 4 |
| Google Scholar | bush babies AND feeding | 7 |
| Google Scholar | galago AND distribution | 138 |
| Google Scholar | galagos AND distribution | 36 |
| Google Scholar | bushbaby AND distribution | 33 |
| Google Scholar | bushbabies AND distribution | 27 |
| Google Scholar | bush baby AND distribution | 11 |
| Google Scholar | bush babies AND distribution | 11 |
|  |  |  |
| Total |  | 2398 (758 without duplicates) |
