# Supplementary material for: Taxonomic and geographic bias in 50 years of research on the behaviour and ecology of galagids
Source: PLoS One. 2021 Dec 15;16(12):e0261379. doi: 10.1371/journal.pone.0261379 (PMC8673608; doi:10.1371/journal.pone.0261379)
Supplement: S2 Table — (DOCX) [file pone.0261379.s002.docx]

***Supplementary Table 2.*** *The 30 most common stemmed words and corresponding un-stemmed words relating to galagid behaviour and ecology, used in scientific papers from January 1971 and December 2020.*

| **Stemmed word** | **Combination of words included** |
| --- | --- |
| behaviour | behave / behaved/ behavior / behavioral / behaviorally / behaviors / behavior-specific / behaviour / behavioural / behaviours |
| area | area / areas |
| pattern | pattern / patterns |
| female | female / females |
| relative | relative / relatives |
| muscle | muscle / muscles / muscle-force / muscle-induced |
| activ* | active / activity / activities / activity / activity-dependent |
| nocturnal | nocturnal / nocturnal-diurnal / nocturnality |
| movement | movement / movements |
| size | size / sizes / sized / size-matched |
| group | group / groups / grouped / grouping / groupings |
| morpholog | morpohlogic / morphological / morphologically / morphology / morphologies |
| bodi | body / bodies |
| cortex | cortex |
| male | male / males / male-female |
| population | population / populations |
| observ | observation / observational / observations / observe / observed / observer / observing |
| function | function / functional / functionally / function-altering / functions |
| region | region / regional / regions |
| visual | visual / visually |
| social | social / sociality / socially |
| range | range / ranged / ranges |
| structur | structural / structure / structures |
| adult | adult / adulthood / adult-like / adults |
| individual | individual / individualized / individually / individuals |
| site | site / sites |
| force | force / forceful / force-production / forces |
| lateral | lateral / lateralis / lateralised / laterality / lateralization / lateralized / laterally |
| period | period / periodically / periods |
| forest | forest / forest-agricultural / forests |
