# Supplementary material for: Taxonomic and geographic bias in 50 years of research on the behaviour and ecology of galagids
Source: PLoS One. 2021 Dec 15;16(12):e0261379. doi: 10.1371/journal.pone.0261379 (PMC8673608; doi:10.1371/journal.pone.0261379)
Supplement: S3 Table — Covariates are: mean annual temperature (‘temperature’;°C); mean human population density from the year 2000 (‘human population density’; humans/km2); and mean annual precipitation (‘precipitation’; ml). Our best model is in bold. (DOCX) [file pone.0261379.s003.docx]

***Supplementary Table 3.*** *Comparison of model performance (AIC) of logistic regression models used to investigate geographic bias in the locations of study sites used to research galagids between January 1971 and December 2020. Covariates are: mean annual temperature (‘temperature’*; °C); mean human population density from the year 2000 (‘human population density’; humans/km^2^); and mean annual precipitation (‘precipitation’; ml). Our best model is in bold.

| **Covariates** | **AIC** |
| --- | --- |
| temperature | 445.98 |
| human population density | 455.98 |
| precipitation | 477.48 |
| **temperature + human population density** | **426.50** |
| temperature + precipitation | 445.00 |
| human population density + precipitation | 457.86 |
| temperature + human population density + precipitation | 428.26 |
